# Nonivamide Enhances miRNA let‐7d Expression and Decreases Adipogenesis PPARγ Expression in 3T3‐L1 Cells

**DOI:** 10.1002/jcb.25052

**Published:** 2015-04-10

**Authors:** Barbara Rohm, Ann‐Katrin Holik, Nicole Kretschy, Mark M. Somoza, Jakob P. Ley, Sabine Widder, Gerhard E. Krammer, Doris Marko, Veronika Somoza

**Affiliations:** ^1^ Christian Doppler Laboratory for Bioactive Aroma CompoundsAlthanstraße 141090ViennaAustria; ^2^ Department of Nutritional and Physiological ChemistryAlthanstraße 141090ViennaAustria; ^3^ Department of Inorganic ChemistryUniversity of ViennaWähringer Straße 42ViennaAustria; ^4^ Symrise AGMühlenfeldstraßeHolzmindenGermany; ^5^ Department of Food Chemistry and ToxicologyUniversity of ViennaWähringer Straße 38ViennaAustria

**Keywords:** *trans‐tert‐*BUTYLCYCLOHEXANOL, LIPID ACCUMULATION, 3T3‐L1 ADIPOGENESIS, CELL DIFFERENTIATION, microRNA, PEROXISOME PROLIFERATOR‐ACTIVATED RECEPTOR (PPAR), TRPV1

## Abstract

Red pepper and its major pungent principle, capsaicin (CAP), have been shown to be effective anti‐obesity agents by reducing energy intake, enhancing energy metabolism, decreasing serum triacylglycerol content, and inhibiting adipogenesis via activation of the transient receptor potential cation channel subfamily V member 1 (TRPV1). However, the binding of CAP to the TRPV1 receptor is also responsible for its pungent sensation, strongly limiting its dietary intake. Here, the effects of a less pungent structural CAP‐analog, nonivamide, on adipogenesis and underlying mechanisms in 3T3‐L1 cells were studied. Nonivamide was found to reduce mean lipid accumulation, a marker of adipogenesis, to a similar extent as CAP, up to 10.4% (*P* < 0.001). Blockage of the TRPV1 receptor with the specific inhibitor *trans*‐*tert*‐butylcyclohexanol revealed that the anti‐adipogenic activity of nonivamide depends, as with CAP, on TRPV1 receptor activation. In addition, in cells treated with nonivamide during adipogenesis, protein levels of the pro‐adipogenic transcription factor peroxisome‐proliferator activated receptor γ (PPARγ) decreased. Results from miRNA microarrays and digital droplet PCR analysis demonstrated an increase in the expression of the miRNA mmu‐let‐7d‐5p, which has been associated with decreased PPARγ levels. J. Cell. Biochem. 116: 1153–1163, 2015. © 2015 The Authors. *Journal of Cellular Biochemistry* published by Wiley Periodicals, Inc.

**Abbreviations**
BCH: *trans*‐*tert*‐butycyclohexanol
miRNA: microRNA
TRPV1: transient receptor potential cation channel subfamily V member 1

Adipose tissue plays a key role in metabolic homeostasis via secretion of adipokines, which interact with central and peripheral organs [Harwood, 2012]. Pathophysiological overgrowth of adipose tissue is associated with overweight, obesity, and subsequent diseases like diabetes type II, chronic inflammation, dementia, and macrovascular diseases [Kivipelto et al., 2005; Wahlqvist, 2005]. One possible mean to regulate total fat mass is to reduce adipogenesis [Bray and Tartaglia, 2000], the differentiation of pre‐adipocytes to mature adipocytes [Hausman et al., 2001], which determines the total number of adipocytes. This process starts during embryonic development, and white adipose tissue largely expands postnatal [Poissonnet et al., 1984]. However, adults are also capable of adipogenesis [Hausman et al., 2001]; about 10% of adipocytes are renewed per year, [Spalding et al., 2008] making the regulation of adipogenesis an interesting target in body weight maintenance.

Capsaicin (CAP), the most abundant capsaicinoid in red pepper, has been shown to be an effective anti‐obesity agent. CAP reduces energy intake [Yoshioka et al., 1999], enhances energy metabolism, and decreases serum triacylglycerol content [Kawada et al., 1986]. In vitro, CAP has been demonstrated to inhibit adipogenesis in 3T3‐L1 cells [Hsu and Yen, 2007], a widely studied in vitro model for the differentiation of pre‐adipocytes to adipocytes. The anti‐adipogenic activity in 3T3‐L1 cells is accompanied by decreased peroxisome‐proliferator activated receptor (PPARγ), C/EPBα, and leptin expression [Hsu and Yen, 2007]. Using transient receptor potential cation channel subfamily V member 1 (TRPV1) deficient 3T3‐L1 cells and knock‐out mice, Zhang et al. [2007] demonstrated that prevention of adipogenesis depends on the activation of the TRPV1. However, binding of CAP to the TRPV1 receptor is also responsible for the pungency of CAP, limiting its dietary intake. This study focuses on the adipogenesis effects of the less pungent CAP‐analog, nonivamide.

Nonivamide, which naturally occurs in *Capsicum oleoresin* as a minor component [Constant et al., 1996], is a direct structural analog of CAP (Fig. 1). It differs from CAP by one methyl group and one double bond on the carbon chain, and exhibits a markedly reduced TRPV1 binding affinity. An EC_50_ value of 0.7 µM for pure CAP has been calculated [Caterina et al., 1997], whereas twice as much of nonivamide is needed for the same effect (EC_50_ = 1.4 µM) [Thomas et al., 2011]. The decrease in TRPV1 binding affinity is accompanied by a major decrease in pungency; pure CAP is rated with 16,000,000 Scoville Heat Units (SHU), whereas nonivamide is rated at 9,200,000 SHUs [Haas et al., 1997]. To investigate the hypothesis, that the less pungent capsaicinoid nonivamide may produce anti‐adipogenic activities similar to those of CAP, lipid accumulation after treatment with CAP and nonivamide was assessed in well‐defined pre‐adipocytes, 3T3‐L1 cells, as a model [Green and Kehinde, 1975]. The process of adipogenesis in 3T3‐L1 cells is well investigated. After reaching confluence, contact inhibition leads to a growth arrest in 3T3‐L1 pre‐adipocytes. A standard hormone cocktail containing insulin, cAMP analogs, and glucocorticoides starts mitotic clonal expansion, involving replication of pre‐adipocytes before terminal differentiation to adipocytes [Gregoire et al., 1998]. This process is regulated by a transcriptional cascade, which involves, but is not limited to peroxisome proliferator‐activated receptor γ (PPARγ), CCAAT‐enhancer binding protein (C/EBP) α, β, and δ and the transcription factors E2F1 and 4 [Rosen and Spiegelman, 2000; Farmer, 2006]. In this process, PPARγ and C/EBPα cross‐activate each other through C/EBP regulatory elements, leading to the transcription of a large group of genes that finally produce the adipocyte phenotype [Clarke et al., 1997]. However, the involvement of several microRNAs (miRNAs) in the regulation of adipogenesis has also been demonstrated [McGregor and Choi, 2011]. miRNAs are small non‐coding RNAs that repress translation and/or promote the decay of its target mRNA by binding to it, hence controlling physiological processes including metabolism, cell proliferation and differentiation [Eulalio et al., 2008]. For instance, the ectopic expression of pro‐adipogenic miR‐103 revealed an up‐regulation of PPARγ_2_, which probably mediates the pro‐adipogenic effects of miR‐103 [Xie et al., 2009]. On the other hand, miR‐27b was shown to directly target PPARγ, whose decreased expression led to an impaired adipogenesis [Karbiener et al., 2009]. However, also miR‐143 and let‐7a have been associated with an increased or decreased, respectively, of PPARγ expression [Esau et al., 2004; Sun et al., 2009].

**Figure 1:**
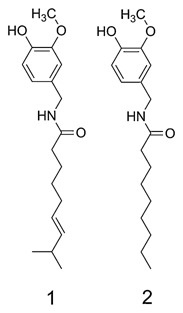
Chemical structures of capsaicin (1) and its analog nonivamide (2).

In order to elucidate mechanisms by which the CAP analog nonivamide may regulate adipogenesis in 3T3‐L1 cells, the dependency of the anti‐adipogenic effects by CAP and nonivamide on TRPV1‐receptor activation was examined using the specific TRPV1‐inhibitor *trans*‐*tert*‐butylcyclohexanol (BCH) [Kueper et al., 2010]. In addition, PPARγ expression, which has previously been described as a target of CAP [Hsu and Yen, 2007], was determined at both the levels of gene expression regulation and protein abundance. To elucidate miRNA involvement in the effect of nonivamide, a genome‐wide miRNA expression analysis was performed by means of a custom‐made microarray. Effects for selected members of the mmu‐let‐7 group were validated using digital droplet PCR.

## MATERIALS AND METHODS

### MATERIALS

Nonivamide and BCH were kindly provided by Symrise AG. Unless stated otherwise, all other chemicals were obtained from Sigma–Aldrich (Austria). Mouse fibroblasts (3T3‐L1) were purchased at ATCC.

### CELL CULTURE

3T3‐L1 pre‐adipocytes cells were maintained in DMEM supplemented with 10% fetal bovine serum, 4% (v/v) L‐glutamine and 1% (v/v) penicillin/streptomycin at 37°C and 5% CO_2_ in a humidified atmosphere. Cells were passaged at ∼75% to 80% confluence and used between passage 6 and 20.

Differentiation into adipocytes was carried out as described before [Riedel et al., 2012]. Briefly, differentiation was initiated 2 days post‐confluence (Day 0) via the addition of differentiation media, consisting of growth medium supplemented with 0.5 mM 3‐isobutyl‐1‐methylxanthine, 1 µM dexamethasone, and 10 µg/ml insulin. After 48 h, differentiation media was replaced by maturation media (DMEM supplemented with 10 µg/ml insulin) on which cells were maintained for a further 48 h. Cells were kept in normal growth media for an additional 5 days. Mature adipocytes were used for experiments on Day 9 after initiation of differentiation. Only monolayers with a differentiation grade of ∼90% or higher were used for the experiments.

The test compounds CAP, nonivamide, and BCH were dissolved in ethanol to 1,000× stock solutions freshly each time and final ethanol concentration during the assays never exceeded 0.2% (v/v).

### MTT ASSAY

Negative effects of a treatment with the test compounds on the number of metabolically active cells were excluded using the MTT assay in 96‐well format. In the MTT assay, the reduction of yellow tetrazolium salt MTT (3‐(4,5‐dimethylthiazol‐2‐yl)‐2,5‐diphenyltetrazolium bromide) to a purple formazan by mitochondrial and ER enzymes is used as a measure for cell viability [Berridge et al., 2005].

Cells were seeded in 96‐well plates and treated with 1 nM–10 µM CAP or nonivamide with or without addition of 25–100 µM BCH or the corresponding ethanol concentration (0.1–0.2% (v/v), solvent control) for 12 days after initiation of differentiation. Cell culture media was exchanged every second day. On Day 12, 100 µl of the MTT working reagent (0.83 mg/ml MTT diluted in PBS/serum‐free media (1:5)), was added to each well, and cells were incubated at 37°C for approximately 15 min. The MTT working solution was removed and the purple formazan formed during incubation was dissolved in 150 µl DMSO per well. Absorbance was measured at 550 nm with 690 nm as reference wavelength using multiwell plate reader (Tecan infinite M200; Tecan Austria). The number of metabolically active cells was calculated relative to untreated control cells or the corresponding solvent control (100%).

### OIL RED O STAINING

Accumulation of lipids was assessed by oil red O staining as described previously [Riedel et al., 2012]. Briefly, 3T3‐L1 pre‐adipocytes were seeded in 24‐well plates at a density of 2 × 10^4^ cells/ml. Cells underwent differentiation as described above, but were maintained in maturation media for 10 days. Substance addition (1 nM–10 µM CAP or nonivamide with or without the addition of 25, 50, or 100 µM BCH, or the corresponding ethanol concentration solely) was started at Day 0 of the induction of differentiation. On Day 12, cells were fixated in 10% (v/v) formalin in PBS for 1 h. Cells were subsequently stained for 10 min with 200 µl oil red O working solution, which contained 21 mg/ml oil red O dye in 60% (v/v) isopropanol. Residual oil red O dye was removed by washing four times with double distilled water. Quantification of the staining was carried out by reading the 520 nm absorbance of the oil red dye from the lipid droplets of the cell monolayer, dissolved in 750 µl isopropanol, on a Tecan infinite M200 multiwell plate reader. Lipid accumulation was calculated as percent of untreated control cells.

### qRT‐PCR

Quantitative Real‐Time PCR was carried out for determination of gene expression levels of PPARγ, C/EBPa, FABP4, and CPT1α. The RNA of 3T3‐L1 cells was extracted on Day 0 (undifferentiated control) and Day 9 after initiation of differentiation with or without compound treatment using the RNeasy Lipid Tissue Mini Kit (Qiagen) according to manufacturer's protocol. Quality and concentration of the RNA was analyzed using the NanoQuant Plate on an infinite M200 Tecan reader. Reverse transcription was carried out using the high capacity cDNA Kit (Life Technologies, Austria). Increasing fluorescence signals during qRT‐PCR reaction were measured in triplicate on a Step‐One Plus device using the Fast SYBR green master mix (Life Technologies). Specific primers for each target gene were designed using NCBI Primer‐BLAST and synthesized by Sigma–Aldrich (Austria) (Table I). Gene expression is given as fold change compared to undifferentiated control cells (=1), calculated from the respective starting mRNA levels, which were determined using LinRegPCR v.12.8 and normalized to hypoxanthine guanine phosphoribosyl transferase (*HPRT1*) as a endogenous control. HPRT1 is a frequently used reference gene for white adipose tissue and 3T3‐L1 cells [Han et al., 2002; Diaz‐Villasenor et al., 2013].

**Table I:** Oligonucleotides Used During PCR Reaction

Target	Forward primer	Reverse primer	Product length (bp)
HPRT	GAGAGCGTTGGGCTTACCTC	ATCGCTAATCACGACGCTGG	136
PPARγ	GTGCCAGTTTCGATCCGTAGA	GGCCAGCATCGTGTAGATGA	142
C/EBPα	GCCCCGTGAGAAAAATGAAGG	ATCCCCAACACCTAAGTCCC	129
FABP4	TTTGGTCACCATCCGGTCAG	TGATGCTCTTCACCTTCCTGTC	110

### PPARγ ELISA

Quantification of PPARγ was carried out using a specific ELISA Kit (mouse PPARγ; Cloud‐Clone Corp., USA) with a sensitivity of 0.66 ng/ml. 3T3‐L1 cells were washed twice with ice‐cold PBS and harvested in lysis buffer (50 mM Tris, 25 mM NaCl, 1 mM EDTA, 1 mM NaF, 1% (v/v) of the non‐denaturing detergent Igepal, pH 7.4) supplemented with 1 mM PMSF, 1 mM sodium *ortho*‐vanadate and protease inhibitor cocktail. Samples were homogenized by passing the lysate several times through a 20‐gauge needle (Sterican, B.Braun Melsungen AG, Germany) and subsequent agitation for 30–45 min at 4°C. The lysate was centrifuged at 16,900*g* for 15 min at 4°C and the PPARγ content in the supernatant quantified by means of the ELISA as recommended by manufacturer's protocol.

### CUSTOMIZED miRNA ARRAY

#### miRNA extraction and labeling

miRNA was extracted using the RNeasy Lipid Tissue Mini Kit (Qiagen) according to the manufacturer's protocol, but exchanging wash buffer RW1 with wash buffer RWT (Qiagen) to preserve RNA pieces <200 bp during washing. RNA quality and concentration was determined with a NanoQuant Plate on an infinite M200 Tecan reader. miRNA was labeled with synthetic 5′‐phosphate‐cytidyl‐uridyl‐DY547‐3′ RNA dinucleotides (Thermo Fisher Scientific) using T4 ligase (New England Biolabs). 300 ng of total RNA (plus synthetic spike‐in controls) were added to the reaction mix containing 1 mM ATP, 50 mM Tris–HCl (pH 7.8), 10 mM MgCl_2_, 1 mM DTT, 10 µg/ml BSA, 25% (v/v) DMSO, 50 µM labeled dinucleotide, and 20 U T4 ligase. The reaction was allowed to take place for 2 h at 4°C and the labeled RNA was purified using a MicroBioSpin 6 column (Bio‐Rad) [Wang et al., 2007].

#### miRNA microarray design and synthesis

Four identical customized micorarrays were synthesized in situ on a glass substrate using a light‐directed maskless array synthesizer as described before [Agbavwe et al., 2011]. The usage of a novel photochemical reaction cell allowed the simultaneous synthesis on two glass substrates, creating eight independently hybridizable microarrays at once [Sack et al., 2013].

Probes were designed for all mature mouse miRNA sequences in the Sanger miRNA database (MiRBase release 19). To equalize melting temperatures of the miRNA probes, microarray probes with very high melting temperatures were shortened at the 3′ side. Since sequences homology among miRNA tends to be near the 5′ end, this shortening has only little effect on sequence specificity; second, microarray probes corresponding to miRNA with very low melting temperatures were extended at the 5′ end by the addition of G or 5′‐AG‐3′ to allow pairing with one or two bases of the ligated dinucleotide [Wang et al., 2007].

#### Hybridization and data analysis

Each microarray was hybridized using a custom design adhesive hybridization chamber (SecureSeal, Grace Biolabs) with a separate compartment for each of the microarrays. The purified, labeled miRNA was applied to the microarray chamber in a hybridization solution containing 100 mM MES, 1 M Na^+^, 20 mM EDTA, 0.01% (v/v) Tween20, and 0.06% (w/v) BSA. Hybridization was carried out at 42°C with constant rotation. Microarrays were scanned with a GenePix 4400 microarray scanner (Molecular Devices, USA) and intensity data for each feature were extracted using NimbleScan software. Each hybridization was performed in duplicates and miRNA levels are presented as mean fold change of the two technical replicates compared to those of undifferentiated control cells.

### DIGITAL DROPLET PCR

Absolute concentrations (copies/µl) of mmu‐let‐7a‐5p, mmu‐let‐7b‐3p, mmu‐let‐7d‐5p, mmu‐miR‐143‐3p, and mmu‐miR‐103‐1‐5p were determined using the Bio‐Rad QX200 Droplet Digital PCR System. For this purpose, miRNA was extracted as described under the microarray section. Extracted miRNA from undifferentiated control cells or mature adipocytes treated with the compounds of interest for 9 days, was subsequently reversely transcribed using the TaqMan MicroRNA Reverse Transcription Kit with specific primers for the target miRNA (Life Technologies). PCR reaction was carried out on a C1000 thermocycler (Bio‐Rad) out using droplet PCR supermix (Bio‐Rad) and TaqMan miRNA Assays (Life Technologies) for each target miRNA after partition of the sample into 20,000 single droplets by means of the droplet generator. Per assay and treatment, between 11,300 and 17,800 droplets were analyzed and the absolute concentrations computed with the QuantaSoft software.

### STATISTICAL ANALYSIS

Data are presented as means ± SEM or fold change compared to control cells (±SEM). Except for the microarray experiments, data are calculated from multiple experiments with at least two technical replicates as indicated in the figure or table legends, at which n refers to the number of biological replicates. Outliers were excluded from calculations after performing Nalimov outlier test. Significant differences between multiple treatments (compound and/or concentration) were determined using One‐ or Two‐Way ANOVA with Holm–Sidak post hoc test. Significant differences between two groups were analyzed with Student's *t*‐test and considered to be different at *P* < 0.05. Differences between groups are marked in figures and tables with *P < 0.05, **P < 0.01, and ***P < 0.001.

## RESULTS

### MTT ASSAY

Negative effects of long‐term treatment with any of the test substances (CAP, nonivamide, and BCH) or a combination thereof, on the number of metabolically active cells were excluded using the MTT assay. There was no reduction in the number of metabolically active cells after a treatment with 0.01–10 µM CAP or nonivamide with or without the addition of 25–100 µM BCH for 12 days compared to control cells (One‐Way ANOVA vs. control, *P* > 0.05, data not shown).

### TREATMENT WITH CAPSAICIN AND NONIVAMIDE REDUCES LIPID ACCUMULATION IN 3T3‐L1 ADIPOCYTES

**Figure 2:**
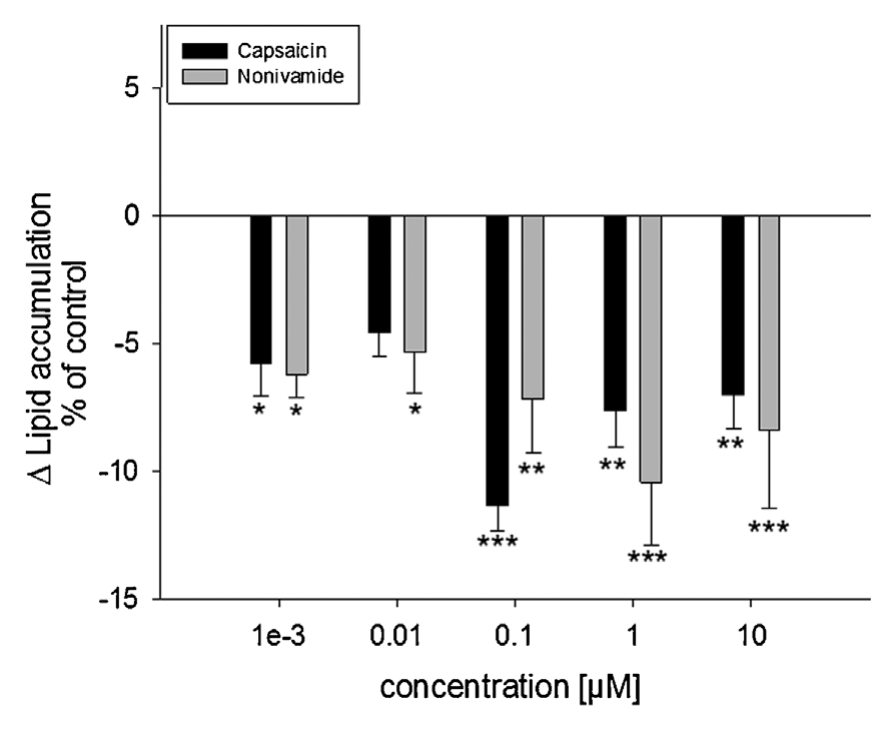
Difference in lipid accumulation in % of control (0.1% EtOH) ± SEM after addition of 0.01 10 µM capsaicin or nonivamide during differentiation and maturation of 3T3‐L1 cells. Lipids in fully mature adipocytes were stained 12 days after initiation of differentiation with oil red O and data are shown as means of control treated cells from three to four independent experiments with at least three technical replicates each. *P < 0.05, **P < 0.01, ***P < 0.001 versus control treated cells.

Accumulation of lipids during the differentiation process, assessed via oil red O staining, is a frequently used functional marker for the degree of adipogenesis in 3T3‐L1 cells [Hwang et al., 2005; Hsu and Yen, 2007; Zhang et al., 2007; Arumugam et al., 2008]. In the present study, the effect of the addition of 0.01–10 µM CAP or nonivamide during differentiation and maturation (12 days) of 3T3‐L1 cells on lipid accumulation was assessed (Fig. 2). First, an effect of addition of 0.1% ethanol as a solvent control to the media was excluded (*P* > 0.05, data not shown). The effects of nonivamide and CAP are, thus, presented compared to cells treated with the solvent control. CAP reduced lipid accumulation by 5.76 ± 1.03% (*P* < 0.05) at 0.01 µM up to 10.1 ± 1.50% (*P* < 0.001) at 0.1 µM in comparison to control cells. Treatment with nonivamide reduced lipid accumulation to a similar extent as CAP; the effects were not different from the effects after CAP treatment at any of the tested concentrations. Compared to untreated control cells, treatment with nonivamide decreased lipid accumulation by 5.34 ± 1.03% (*P* < 0.05) at 0.01 µM up to 10.4 ± 2.47% (*P* < 0.001) at 1 µM.

### REDUCTION IN LIPID ACCUMULATION BY CAPSAICIN AND NONIVAMIDE CAN BE BLOCKED BY THE ADDITION OF A TRPV1 INHIBITOR

**Figure 3:**
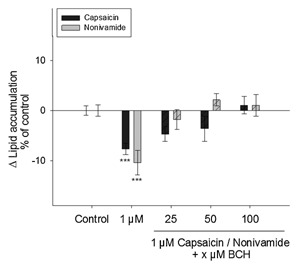
Difference in lipid accumulation in % of control (0.1% EtOH) ± SEM after treatment with 1 µM capsaicin or nonivamide with or without the addition of 25–100 µM of the selective TRPV1 inhibitor *trans*‐*tert*‐butylcyclohexanol (BCH) during differentiation and maturation (12 days) of 3T3‐L1 cells. Lipids in fully mature adipocytes were stained 12 days after initiation of differentiation with oil red O and data are shown as means compared to control treated cells from three to four independent experiments with at least three technical replicates each. ***P < 0.001 versus control.

Activation of the TRPV1 receptor has been shown to be responsible for the anti‐adipogenic effects of CAP in vitro and in vivo [Zhang et al., 2007]. In order to examine whether the effects of nonivamide on lipid accumulation also depend on TRPV1 activation, 3T3‐L1 cells were co‐incubated with 1 µM nonivamide and 25–100 µM of the specific TRPV1‐inhibitor BCH during differentiation and maturation for a total of 12 days (Fig. 3). A concentration of 1 µM nonivamide was chosen for co‐incubation studies, since this concentration demonstrated the greatest effect. As a positive control for TRPV1 inhibition by BCH, the effect of concomitant incubation of the TRPV1 inhibitor BCH and CAP was determined. Addition of BCH to CAP‐containing media prevented reduction in lipid accumulation by CAP, leading to no difference between control treatment and a treatment with 1 µM CAP plus 25 µM (−4.71 ± 1.45%), 50 µM (−3.62 ± 2.49%) and 100 µM BCH (+1.06 ± 1.73%, *P* < 0.05 vs. control), whereas treatment with 1 µM CAP alone reduced lipid accumulation by 7.63 ± 1.41% (*P* < 0.001, Fig. 3). Likewise, addition of 25, 50, and 100 µM BCH to media containing 1 µM nonivamide prevented reduction in lipid accumulation caused by 1 µM nonivamide (−10.4 ± 2.47%, *P* < 0.001 vs. control, Fig. 3) as well. There was, similarly to the results obtained for CAP, no difference between control‐treated cells and cells treated with 25 µM (−1.83 ± 2.00%), 50 (+2.15 ± 1.25%), and 100 µM BCH (+1.0 ± 2.18%) in combination with nonivamide (*P* > 0.05 for each treatment vs. control, Fig. 3). Incubation of 3T3‐L1 cells for 12 days during differentiation and maturation with 25–100 µM BCH did not affect lipid accumulation compared to control cells (*P* > 0.05) and was between 2.07 ± 1.35% and −0.96 ± 1.93% (data not shown in figure).

### TREATMENT WITH NONIVAMIDE DECREASES EXPRESSION OF PPARγ

**Figure 4:**
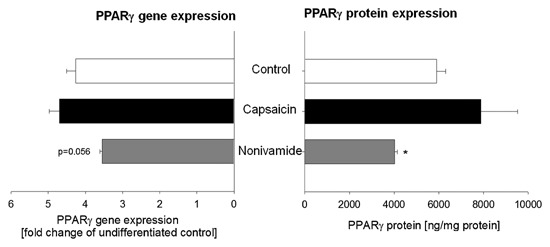
PPARγ expression on genetic level (as mean fold change ± SEM compared to undifferentiated control cells, left side) and protein (in ng/mg protein ± SEM, right side) level in 3T3‐L1 adipocytes. mRNA or protein was extracted on Day 9 after initiation of differentiation. During differentiation and maturation, 3T3‐L1 cells were treated with either 0.1% EtOH, 10 µM capsaicin, or nonivamide. Data are shown as mean from three independent experiments. *P < 0.05 versus control.

PPARγ and C/EBPα are major factors regulating adipogenesis and have been demonstrated as a target of CAP [Hsu and Yen, 2007]. Thus, gene expression levels of PPARγ, C/EBPα, and FABP4 after treatment with 10 µM nonivamide or CAP for 9 days or of control treated cells were determined using qPCR. Compared to undifferentiated control cells, gene expression of PPARγ, C/EBPα, and FABP4 increased to 4.26 ± 0.25, 7.51 ± 0.43, and 153 ± 10.2 in control treated cells (Fig. 4, Table II). However, there was no significant impact of CAP or nonivamide treatment on gene expression of C/EBPα and FABP4 and PPARγ, although there was a trend (*P* = 0.056) toward a down‐regulation of PPARγ mRNA levels after treatment with nonivamide (3.55 ± 0.06, Fig. 4, left side).

**Table II:** Results of the Gene Expression Analysis of C/EBPα and FABP4

Target gene	Solvent control	Capsaicin	Nonivamide
*C/EBPα*	7.51 ± 0.43	7.69 ± 0.59	6.28 ± 0.33
*FABP4*	153 ± 10.2	155 ± 21.3	144 ± 9.78

Data are shown as fold changes compared to undifferentiated control cells (=1) from three independent experiments with at least two technical replicates. mRNA for the experiments was extracted on Day 0 (undifferentiated control) and Day 9 after initiation of differentiation, during which cells were either treated with 0.1% EtOH or 10 µM capsaicin or nonivamide.

Fold changes in gene expression after treatment with 0.1% EtOH (solvent control) or 10 µM capsaicin or nonivamide compared to undifferentiated control cells (=1). n = 3 with three technical replicates.

In order to investigate whether PPARγ levels are down‐regulated at the protein level, the PPARγ content per mg protein of 3T3‐L1 cell lysates 9 days after initiation of differentiation with or without treatment with CAP or nonivamide was determined. Undifferentiated control cells had an average PPARγ content of 59 ± 16.7 ng/mg protein. Upon differentiation, PPARγ levels increased by a factor of 132, to 5894 ± 416.6 ng/mg protein (*P* < 0.001) in control treated cells. Treatment with CAP did not change PPARγ levels (7882 ± 3654 ng/mg protein) compared to those of control treated cells, whereas treatment with nonivamide led to a decrease of PPARγ to 4016 ± 116 ng/mg protein compared to control treated cells (*P* < 0.05, Fig. 4, right side).

### TREATMENT WITH NONIVAMIDE REGULATES EXPRESSION OF mmu‐let‐7d‐5p

Since several miRNAs have been associated with the regulation of adipogenesis and obesity (see review: [McGregor and Choi, 2011]), a genome‐wide miRNA array was performed. By means of this customized array, miRNA levels of undifferentiated 3T3‐L1 cells, control treated cells (0.1% EtOH) and cells treated with 10 µM nonivamide during adipogenesis for 9 days were compared. Fold changes of control and nonivamide‐treated cells compared to undifferentiated cells of selected miRNAs, which have been associated with the regulation of adipogenesis before, are displayed in Table III. On Day 9 after initiation of adipogenesis, expression levels of mmu‐miR‐103‐3p (4.35), mmu‐miR‐210‐3p (1.73), as well as mmu‐let‐7a‐5p (2.04), and mmu‐let‐7d‐5p (1.82) were increased compared to undifferentiated control cells (=1), using an absolute fold change of 1.5 as cut‐off criteria [Li et al., 2011]. In contrast, there was neither an effect on other isoforms of the upregulated miRNAs, nor on further adipogenesis‐regulating miRNAs like mmu‐miR‐143, mmu‐miR‐193, mmu‐miR‐27, or mmu‐miR‐448. However, treatment with nonivamide for 9 days increased expression of mmu‐let‐7a‐5p from 2.04 (control treatment/undifferentiated control) to 3.38 (nonivamide treatment/undifferentiated control), corresponding to an absolute fold change of 1.66. Also other members of the let‐7 group were up‐regulated after nonivamide treatment, leading to an increased expression of mmu‐let‐7b‐3p from 1.10 to 3.77, corresponding to an absolute fold change of 1.66, and mmu‐let‐7d‐5p from 1.82 to 2.73, corresponding to an absolute fold change of 1.5. In contrast, mmu‐miR‐103‐1‐5p and mmu‐miR‐103‐2‐5p were down‐regulated after nonivamide treatment to 0.06 and 0.25 compared to 1.16 and 0.93 after control treatment. Nonivamide‐treatment also reduced expression of mmu‐miR‐143‐3p (0.06), mmu‐miR‐210‐3p (0.14), mmu‐miR‐27a‐3p and ‐5p to 0.17 or 0.09, respectively, and mmu‐miR‐27b‐5p to 0.19 (Table III).

**Table III:** Results of the Customized miRNA Microarray Using an Absolute Fold Change (Compared to Undifferentiated Control Cells) of 0.5 or 1.5, Respectively, as Cut‐Off Criteria

Mature miRNA	Solvent control	Nonivamide
mmu‐let‐7a‐5p	2.04	3.38
mmu‐let‐7b‐3p	1.10	3.77
mmu‐let‐7d‐5p	1.82	2.73
mmu‐miR‐103‐3p	4.35	6.37
mmu‐miR‐103‐1‐5p	1.16	0.06
mmu‐miR‐103‐2‐5p	0.93	0.25
mmu‐miR‐143‐3p	1.29	0.06
mmu‐miR‐193a‐3p	1.00	0.09
mmu‐miR‐193a‐5p	0.94	0.01
mmu‐miR‐193b‐5p	0.88	0.26
mmu‐miR‐27a‐3p	1.03	0.17
mmu‐miR‐27a‐5p	1.04	0.09
mmu‐miR‐27b‐5p	0.79	0.19
mmu‐miR‐210‐3p	1.73	0.14
mmu‐miR‐448‐3p	0.79	0.02
mmu‐miR‐448‐5p	0.81	0.10

Data are shown as fold changes compared to undifferentiated control cells (=1) of selected miRNAs, that were shown to regulate adipogenesis in 3T3‐L1 cells. miRNA for the experiments was extracted on Day 0 (undifferentiated control) and Day 9 after initiation of differentiation, during which cells were either treated with 0.1% EtOH or 10 µM nonivamide.

Fold changes in miRNA expression after treatment with 0.1% EtOH (solvent control) or 10 µM nonivamide compared to undifferentiated control cells (=1). n = 1 with two technical replicates.

**Figure 5:**
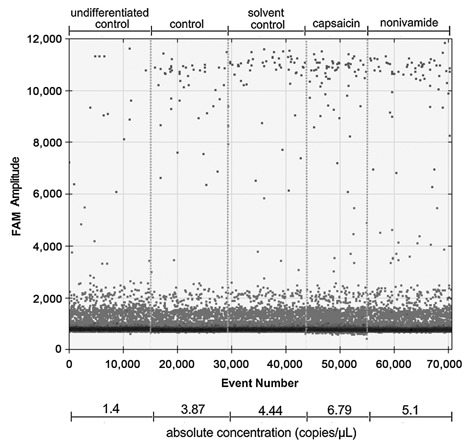
Visual representation of one example measurement of the weakly expressed mmu‐let‐7b‐3p using ddPCR. The x‐axis shows the accumulating number of counted droplets. Per treatment, between 15,024 (capsaicin) and 16,357 (solvent control) droplets were accepted for analysis of a negative or positive FAM signal. FAM signal intensity is displayed on the y‐axis. The lower cluster represents negative droplets, whereas the upper cluster represents droplets with a positive signal, allowing calculation of absolute copy numbers (copies/µl) using QuantaSoft software.

**Figure 6:**
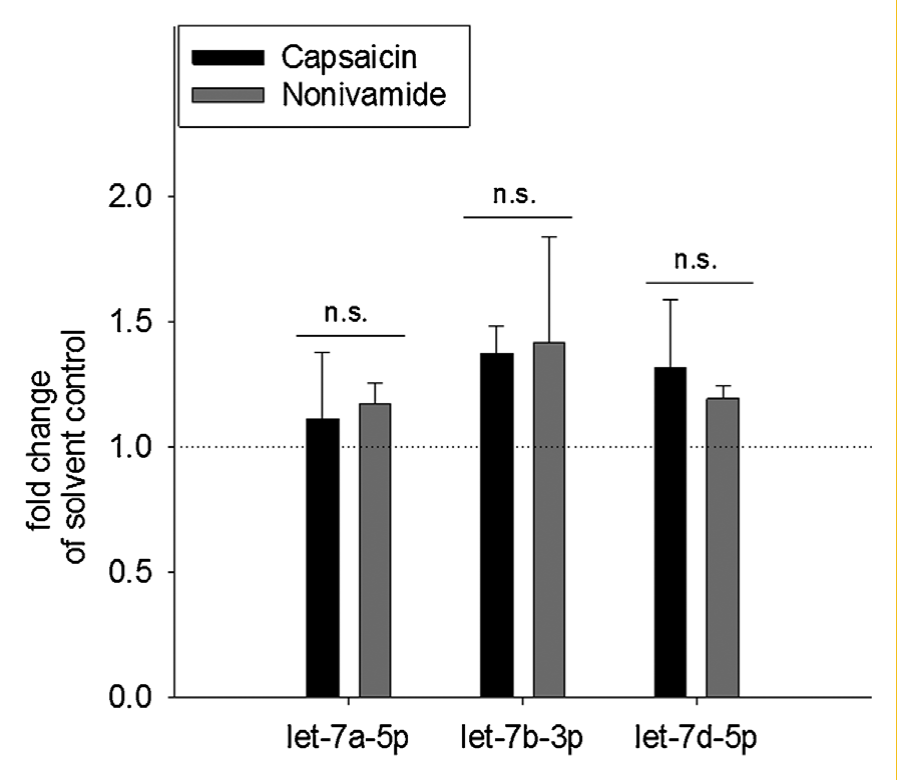
Mean fold changes in mmu‐let‐7a‐5p, mmu‐let‐7b‐3p, and mmu‐let‐7d‐5p expression were analyzed using digital droplet PCR. miRNA from 3T3‐L1 cells was extracted on Day 9 after initiation of differentiation. During the process of differentiation and maturation, cells were treated with either 10 µM capsaicin or nonivamide, or the corresponding ethanol concentration (0.1% ethanol; solvent control). An effect of the ethanol treatment was excluded. Data are displayed as mean fold changes ± SEM compared to the solvent control (=1, dotted line) of three independent experiments.

Since the present study detected a similar expression pattern after nonivamide treatment for several members of mmu‐let‐7, the expression of selected isoforms was validated using highly sensitive digital droplet PCR. This methods allows an absolute quantification of the target gene copy number per µl by partition of the 20 µl test sample into 20,000 single droplets that are separately analyzed for a positive or negative fluorescence signal (Fig. 5). Compared to undifferentiated control cells, expression of mmu‐let‐7a‐5p, mmu‐let‐7b‐3p, and mmu‐let‐7d‐5p was increased to a fold change of 1.44 ± 0.07, 5.91 ± 0.83, or 2.22 ± 0.19, respectively, within 9 days after initiation of differentiation in control cells. Treatment with the solvent control 0.1% EtOH during differentiation led to similar fold changes compared to undifferentiated cells with 1.47 ± 0.30 for mmu‐let‐7a‐5p, 5.86 ± 0.43 for mmu‐let‐7b‐3p, and 2.47 ± 0.28 for mmu‐let‐7d‐5p (*P* > 0.05). In contrast to the miRNA array results, mmu‐let‐7a‐5p expression was not affected by treatment with CAP or nonivamide with fold changes to the solvent control of 1.11 ± 0.26 or 1.18 ± 0.08, respectively (Fig. 6). However, expression of mmu‐let‐7b‐3p increased to 8.05 ± 0.64 in CAP‐treated cells compared to the solvent control (5.86 ± 0.43, *P* < 0.05), corresponding to a fold change of 1.38 ± 0.11 (Fig. 6). Treatment with nonivamide increased mmu‐let‐7b‐3p expression to a comparably mean fold change of 8.32 ± 2.46 (1.42 ± 0.42 compared to the solvent control), without reaching the level of significance (*P* > 0.05). Expression of mmu‐let‐7d‐5p increased (2.95 ± 0.0.13) in nonivamide‐treated cells compared to control cells (2.22 ± 0.19, *P* < 0.01), but not compared to the solvent control (corresponding fold change 1.20 ± 0.05, *P* > 0.05). Treatment with CAP led to a similar fold change of undifferentiated control cells of 3.25 ± 0.17, corresponding to a fold change of 1.32 ± 0.28 of the solvent control, without reaching the level of significance (*P* > 0.05). No difference in mmu‐let‐7 expression in response to CAP and nonivamide‐treatment was found (Fig. 6).

## DISCUSSION

Red pepper and its major pungent principle, CAP are often discussed as anti‐obesity agents. Beside reducing energy intake [Yoshioka et al., 1999], increasing energy metabolism and lowering serum triacylglycerol content [Kawada et al., 1986], administration of 0.01% (w/w) CAP has been shown to reduce visceral adipose tissue and subcutaneous fat in mice fed a high fat diet [Zhang et al., 2007]. In addition, CAP has been shown to reduce adipogenesis in 3T3‐L1 pre‐adipocytes [Hsu and Yen, 2007; Zhang et al., 2007]. Knock out experiments in in vitro and in vivo model systems have shown that anti‐adipogenic activity of CAP is mediated by activation of the TRPV1 cation channel [Zhang et al., 2007]. However, the downside of CAP, being a highly potential TRPV1 agonist, is that its contact with mucous membranes, for example, in the oral cavity, leads to a sharp burning pain in mammals. This pungency strongly limits dietary intake of CAP, especially in European countries. In the present study, we investigated whether the less pungent capsaicinoid, nonivamide, may exhibit similar effects on adipogenesis in 3T3‐L1 cells as CAP. Nonivamide is a direct structural analog of CAP, although the slight structural difference reduces its TRPV1 binding affinity and hence, also its pungency by half. In the present study, we analyzed lipid accumulation by oil red O staining, as an indicator for adipogenesis. Oil red O staining is a frequently used marker for differentiation of pre‐adipocytes to adipocytes in 3T3‐L1 cells [Arumugam et al., 2008; Yoshitomia et al., 2012; Zhang et al., 2011]. Beside the visible accumulation of lipid droplets, the strong increase compared to undifferentiated control cells in PPARγ gene and protein expression as well as C/EBPα and FABP4 gene expression further confirmed the differentiation of 3T3‐L1 pre‐adipocytes to mature adipocytes upon addition of the hormone cocktail 2 days post‐confluence.

The results demonstrate that addition of 0.01–10 µM nonivamide reduces lipid accumulation in 3T3‐L1 cells up to 10.4 ± 2.47% when added at a final concentration of 1 µM during differentiation and maturation for 12 days, which is comparable to the results obtained for CAP. Treatment with CAP reduced lipid accumulation up to 10.1 ± 1.50% at 0.1 µM, confirming the results of Zhang et al. [Hsu and Yen, 2007; Zhang et al., 2007], who showed reduced oil red O staining of 3T3‐L1 cells after treatment with 1 µM of CAP during adipogenesis. In order to investigate whether the nonivamide‐induced reduction in lipid accumulation is mediated via TRPV1 activation, the effect of concomitant addition of the selective TRPV1 inhibitor BCH and nonivamide on lipid accumulation was analyzed. BCH has been successfully used as a TRPV1‐inhibitor in previous studies [Rohm et al., 2013, 2015]. Since the anti‐adipogenic effect of CAP depends on TRPV1 receptor activation [Hsu and Yen, 2007; Zhang et al., 2007], the effect of BCH on the reduction of lipid accumulation by CAP was used as a positive control for TRPV1 blockage by BCH. In the presence of 25–100 µM BCH, addition of 1 µM CAP did not reduce lipid accumulation, proving the effectiveness of BCH and confirming previous results [Hsu and Yen, 2007; Zhang et al., 2007]. However, addition of 25–100 µM BCH to nonivamide‐containing media prevented the anti‐adipogenic activity of nonivamide, leading to no reduction in lipid accumulation compared to control treated cells. This result demonstrates that activation of the TRPV1 receptor by both CAP and nonivamide inhibits adipogenesis in 3T3‐L1 cells. However, since ethanol has also been discussed to activate the TRPV1 receptor [Blednov and Harris, 2009; Trevisani et al., 2002], an effect of low doses of ethanol as solvent (0.1–0.2%) for the test substances on lipid accumulation was excluded in preliminary experiments.

As a signaling pathway for TRPV1‐mediated inhibition of adipogenesis, increased calcium entry from the extracellular space via the TRPV1 channel with intracellular calcium accumulation targets adjacent calcineurin [Cioffi, 2007]. Activation of calcineurin is thought to inhibit the pro‐adipogenic factors PPARγ and C/EBPα, thus repressing adipogenesis [Cioffi, 2007]. This suggested pathway is supported by a study from Hsu and Yen [2007], who demonstrated that treatment of mature 3T3‐L1 adipocytes with high concentrations (25–100 µM) CAP for 12–24 h down‐regulated expression of PPARγ and C/EBPα. Thus, we investigated the effect of CAP and nonivamide treatment during adipogenesis on gene expression of C/EBPα and PPARγ, and, as a further marker for adipogenesis, FABP4. Gene expression of the three markers increased during adipogenesis, although there was no effect of CAP and nonivamide treatment compared to control treated cells. Since there was a trend (*P* = 0.056) toward a PPARγ down‐regulation after nonivamide treatment, PPARγ protein expression was analyzed as well. In contrast to CAP exposure, nonivamide‐treatment reduced PPARγ expression compared to control‐treated cells. This down‐regulation of PPARγ could at least partly account for inhibition of adipogenesis by nonivamide. Although the comparable anti‐adipogenic activities of CAP and nonivamide both depend on TRPV1 activation, treatment with CAP did not affect PPARγ treatment, contrary to the hypothesis and existing evidence from the literature. However, down‐regulation of PPARγ after CAP treatment in the study by Hsu et al. was observed after treatment with far higher concentrations of CAP (25–100 µM). In addition, in the study by Hsu and Yen [2007], mature 3T3‐L1 adipocytes were treated, whereas in the present study, 3T3‐L1 cells were treated during differentiation process. Also, a counter‐regulation of other genes cannot be excluded. However, the differences in PPARγ expression between CAP and nonivamide treatment are unexpected, and point to possible differences in signaling pathways. Differences in signaling after CAP and nonivamide treatment have been shown before in neural SH‐SY5Y cells [Rohm et al., 2013], and can hence not be excluded for the present study as well. In addition, it remains to be clarified whether the differences in PPARγ expression after treatment with nonivamide or CAP originate at the pre‐ or at the post‐transcriptional level.

The pro‐adipogenic transcription factor PPARγ has been shown to be a target of some miRNAs, which have been recently identified as a novel group of adipogenic regulators. To investigate, whether the anti‐adipogenic activity of nonivamide involves, beside TRPV1 activation and PPARγ down‐regulation, also a regulation of miRNAs, a customized miRNA microarray was carried out for a first screening. During adipogenesis, miRNA‐103‐3p, miR‐210‐3p, and let‐7a‐5p, let‐7b‐3p, and let‐7d‐5p expression increased compared to undifferentiated control cells. This is in accordance with previous studies [Sun et al., 2009; Xie et al., 2009; Liang et al., 2013], although other isoforms of the presented miRNAs were not regulated.

**Figure 7:**
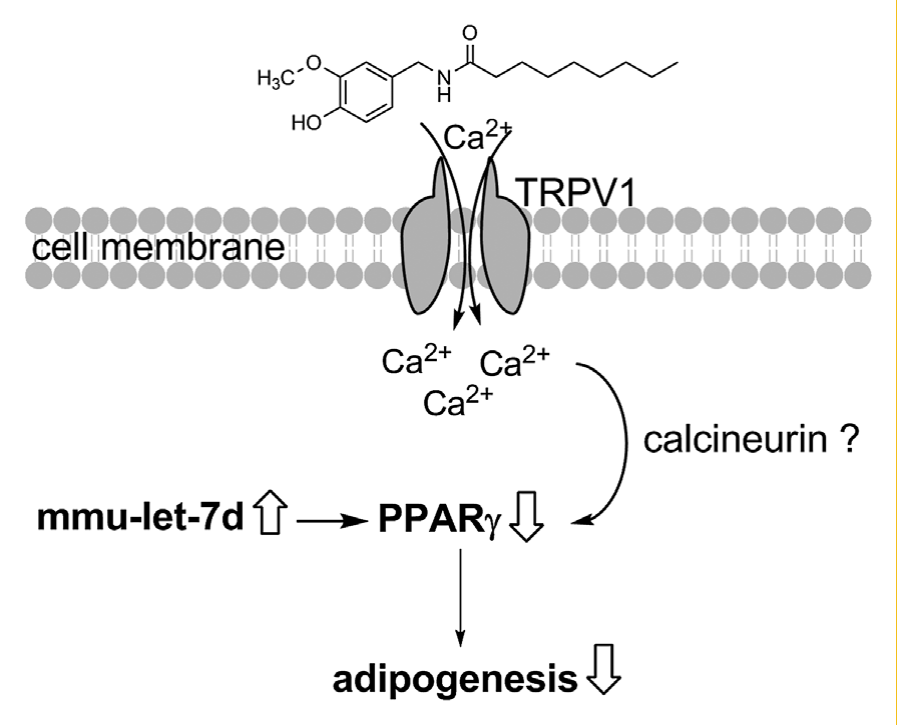
Hypothesized pathway for the anti‐adipogenic activity of nonivamide in 3T3‐L1 cells. Binding of nonivamide to the TRPV1 cation channel increases intracellular Ca^2+^, which decreases expression of PPARγ, possibly via calcineurin. In addition, increased expression of the miRNA mmu‐let‐7d might decrease PPARγ expression as well, impairing differentiation of 3T3‐L1 cells to an adipocyte phenotype.

Treatment with nonivamide led to a down‐regulation of miR‐27a‐3p/‐5p and miR‐27b‐5p compared to control treatment, which would rather argue for a PPARγ up‐regulation than the analyzed PPARγ down‐regulation after nonivamide treatment [Karbiener et al., 2009]. In contrast, the detected down‐regulation of miR‐143‐3p after nonivamide treatment compared to control treatment could at least partly explain inhibition of PPARγ expression [Esau et al., 2004]. Also expression levels of the anti‐adipogenic mmu‐let‐7a‐5p, mmu‐let‐7b‐3p, and mmu‐let‐7d‐5p increased after nonivamide treatment compared to control treatment, which has been associated with an decreased PPARγ expression before [Sun et al., 2009]. Changes in expression of mmu‐let‐7a‐5p, mmu‐let‐7b‐3p, and mmu‐let‐7d‐5p were validated using ddPCR, which allows a much more precise, absolute quantification of the target gene/miRNA than qPCR or microarray [Hindson et al., 2011, 2013]. Absolute quantification of these selected members of the let‐7 group in undifferentiated control cells (Day 0) and 9 days after initiation of differentiation confirmed the results of the microarray by demonstrating an up‐regulation of all three representatives of the let‐7 group during adipogenesis. However, using ddPCR, there was no impact of CAP or nonivamide treatment on mmu‐let‐7a‐5p expression. CAP‐treated cells showed an increased let‐7b‐3p expression compared to solvent control‐treated cells, whereas treatment with nonivamide led to an increased expression of mmu‐let‐7d‐5p compared to the control. An increased expression of mmu‐let‐7d‐5p after nonivamide‐treatment compared to solvent control treated cells was also detected using the customized microarray, validating the stimulating impact of nonivamide treatment on mmu‐let‐7d‐5p expression. Increased expression of let‐7 has been shown to be accompanied by decreased PPARγ expression [Sun et al., 2009]. Thus, increased mmu‐let‐7d‐5p may be responsible for the decreased PPARγ in nonivamide‐treated cells and hence be involved in the anti‐adipogenic activity of nonivamide in 3T3‐L1 cells. Figure 7 provides an overview of the hypothesized signaling pathway for the anti‐adipogenic activity of nonivamide. It is also remarkably that, although treatment with CAP did not reduce PPARγ expression, there was no difference in the expression of the investigated let‐7 representatives between nonivamide and CAP treated cells.

In conclusion, the present study demonstrates for the first time that the less pungent CAP‐analog nonivamide impairs adipogenesis to a similar extent as CAP. Using a specific inhibitor, it was demonstrated that the anti‐adipogenic activity of nonivamide depends, like the anti‐adipogenic activity of CAP, on the activation of the TRPV1 receptor. Nonivamide has a lower binding affinity than CAP to the TRPV1 receptor, however, in the tested range of concentrations, effects of nonivamide and CAP on adipogenesis were equal. The effects of lower test concentrations would be needed to clearly identify the activity threshold for both compounds, and to determine whether the threshold can be correlated with TRPV1 binding affinity, and thus, pungency. However, a different downstream‐signaling pathway after TRPV1 activation is conceivable, since contrary to CAP, treatment with nonivamide decreased PPARγ levels. This could, at least partly, be explained by an increased expression of the miRNA mmu‐let‐7d. Since the capsaicinoide nonivamide is rated to almost half as pungent as CAP [Haas et al., 1997], an oral application of higher doses compared to CAP is possible and reveals nonivamide as a less pungent, but still potent novel anti‐obesity compound from nature.

Although data from long‐term human intervention studies with nonivamide are lacking, nonivamide seems to be a promising candidate to target different medicinal strategies in the treatment of obesity. Beside the inhibition of adipogenesis demonstrated here, nonivamide has also been shown to decrease fatty acid uptake in Caco‐2 cells [Rohm et al., 2015], which may support the prevention of hyperlipidemia. In addition, administration of 0.15 mg nonivamide in an oral glucose tolerance test reduced total energy intake from a standardized breakfast in slightly overweight male subjects [Hochkogler et al., 2014], supporting the effectiveness of the less pungent CAP‐analog.
